# Efficacy and long-term longitudinal follow-up of bone marrow mesenchymal cell transplantation therapy in a diabetic patient with recurrent lower limb bullosis diabeticorum

**DOI:** 10.1186/s13287-018-0854-9

**Published:** 2018-04-10

**Authors:** Yan Chen, Yu Ma, Ning Li, Hongyan Wang, Bing Chen, Ziwen Liang, Rui Ren, Debin Lu, Johnson Boey, David G. Armstrong, Wuquan Deng

**Affiliations:** 1 Department of Endocrinology and Nephrology, Diabetic Foot Center, Affliated Central Hospital of Chongqing University, Chongqing Emergency Medical Hospital, Chongqing, China; 2 Department of Endocrinology, Southwest Hospital, Army Medical University, Chongqing, China; 3 Department of Endocrinology, the 9th People’s Hospital of Chongqing, Chongqing, China; 4 Department of Podiatry, Singapore General Hospital, Bukit Merah, Singapore; 5 Department of Surgery, Keck School of Medicine of the University of Southern California, Los Angeles, CA USA

**Keywords:** Diabetes mellitus, Bullosis diabeticorum, Diabetic peripheral arterial disease, Bone marrow mesenchymal stem cells

## Abstract

Bullosis diabeticorum is a rare presentation of cutaneous manifestation most commonly affecting the lower limbs in patients with diabetes. The appearance, often as insidious as its resolution, is characterized by tense blisters on the skin surfaces of the lower limbs and the feet. The cause still remains unclear, but it may relate to microangiopathy and neuropathy. In this report, we present a case of a 64-year-old male with multiple episodes of blistering in the left lateral lower limb after a traumatic fall who was subsequently diagnosed with type 2 diabetes mellitus. The patient had a history of poorly controlled blood glucose and subsequently developed vasculopathy and peripheral neuropathy. Despite appropriate glycemic control and antibiotics therapy, the patient developed recurrent bullosis diabeticorum on five separate occasions during a 2-year span from 2005 to 2007. Building on our success with ischemic diabetic foot, we used bone marrow mesenchymal stem cell (BMMSC) transplantation therapy for bullosis diabeticorum. After a 9-month treatment, this patient developed another episode of cellulitis in the same lower limb which was successfully treated with antibacterial therapy. It is interesting that the patient reported no recurrence in the next 10-year follow-up span. This study demonstrates that bullosis diabeticorum could appear even before the onset of diabetes, and vascular insufficiency predisposes to the occurrence of bullosis diabeticorum. Our findings suggest that autologous BMMSC transplantation therapy may be an effective measure for recurrent bullosis diabeticorum; however, this will require further investigation to be conclusive. Early identification of diabetes and its complications and appropriate treatment may improve clinical outcomes and prevent lower limb amputation.

Trial registration: ClinicalTrials.gov Identifier: NCT00955669. Registered on August 10, 2009.

## Background

**Fig. 1:**
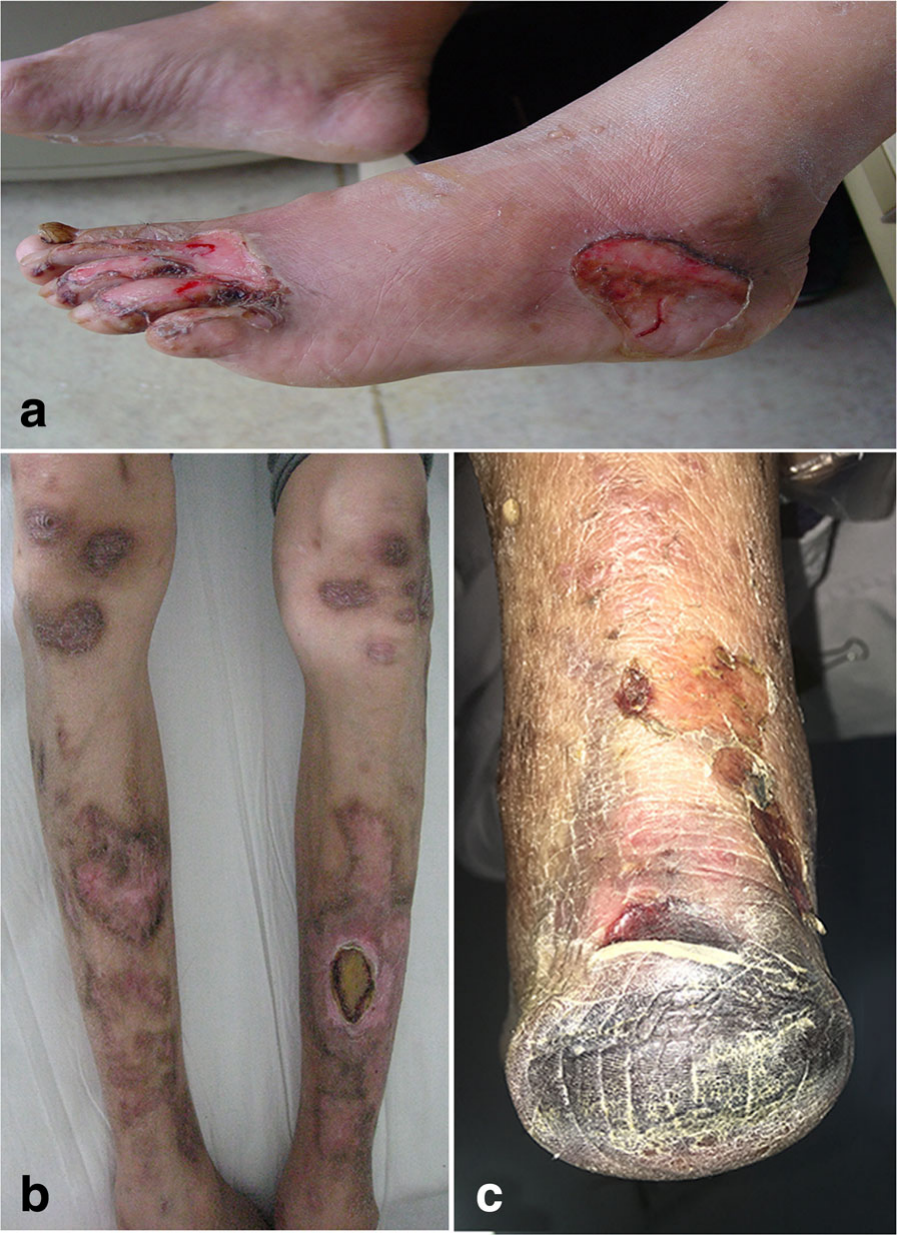
**a** Bullosis diabeticorum with skin ulcer. **b** Bullosis diabticorum with diabetic osteomyelitis. **c** Bullosis diabeticorum with wet gangrene

**Fig. 2:**
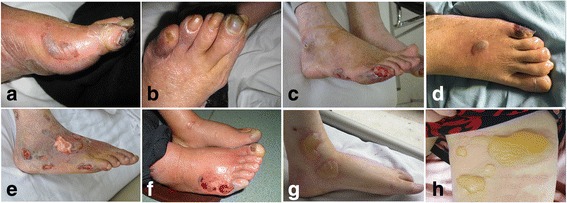
**a**-**d** Ischemic bullosis diabeticorum. **e**-**h** Neuropathic bullosis diabeticorum

Dermatological diseases are relatively common in diabetic patients, in which they manifest cutaneously as a variety of conditions such as bacterial and fungal infections, diabetic dermopathy, granuloma annulare, and necrobiosis lipoidica diabeticorum [1]. Bullosis diabeticorum is a rare cutaneous disease, with 100 cases or case series reported in the literature [2], characterized by spontaneous noninflammatory manifestations, painless subcutaneous fluid-filled vesicles varying in size from a few millimeters to a few centimeters. It is usually distributed in the lower extremities, in which there is an observed risk of developing secondary infection, including diabetic skin ulcer (Fig. 1a), osteomyelitis (Fig. 1b), or wet gangrene (Fig. 1c), even diabetic amputation. Presently the exact etiology of bullosis diabticorum is not well understood. Several studies revealed that its occurrence is closely related to diabetic patients with complications of microangiopathy [3], neuropathy, and poor regulation of blood glucose [4]. Bullosis diabeticorum is not uncommon in our clinical experience and observation; an average of 250 people with diabetes per year with foot problems (skin disease, ulcer, gangrene) were treated in the past 10 years, including about 60 cases with bullosis diabticorm. Thus, there is a bullosis diabeticorum incidence rate of 2.4% per year in our clinic. Bullosis diabticorum is prone to occur in patients with local microcirculation dysfunction (Fig. 2a-d) and diabetic neuropathy (Fig. 2e-h). Often, conservative treatment is often considered, while aggressive surgical debridement and subsequently skin grafting are indicated in more severe cases [5]. Moreover, the elimination of causative factors is imperative for prevention of its recurrence and complications [4, 5]. In our previous study, we successfully treated diabetic critical limb ischemia with bone marrow mesenchymal stem cells (BMMSCs) [6, 7]. To further explore their benefits, we have administered BMMSC transplantation therapy to a patient with recurrent bullosis diabeticorum in the left lower limb complicated by limb ischemia and mild venous insufficiency.

## Methods

### Patient

**Fig. 3:**
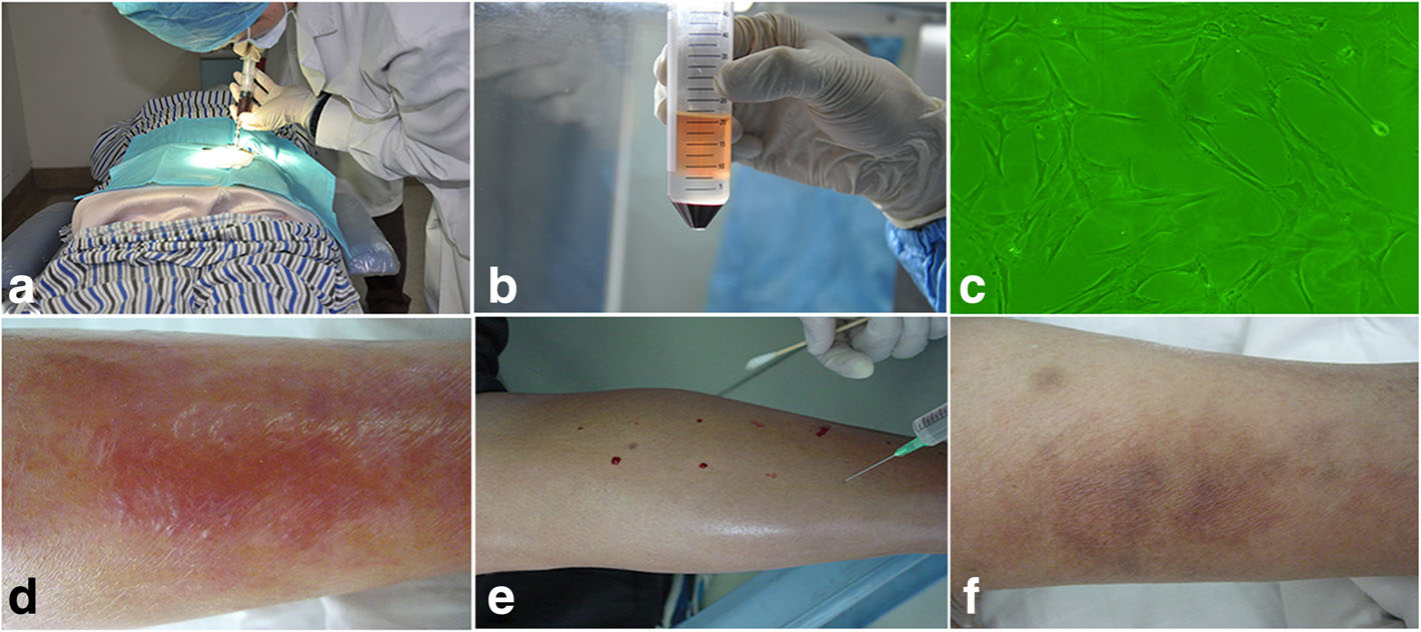
Bone marrow mesenchymal stem cell (BMMSC) transplantation procedure. **a** Extraction of bone marrow under disinfected and anesthetic conditions. **b** The mononuclear stem cell layer was harvested with density gradient centrifugation. **c** BMMSC morphology after 21 days of culture. **d** Subcutaneous cellulitis with bullosis diabeticorum. **e** Administration of BMMSCs with normal saline. **f** The left lower limb 10 years after BMMSC treatment

A 64-year-old male presented to hospital in July 2004 for the sudden occurrence of cutaneous blisters of varying sizes with associated signs and symptoms of cellulitis in the left lateral lower limb, including erythema, edema, increased skin temperature, and tenderness. In May 2005, the patient was re-admitted to the hospital with similar signs and symptoms in the left lower limb when he was diagnosed with diabetes mellitus based on fasting serum glucose level and 2-h postprandial serum glucose (10.06 mmol/L and 14.6 mmol/L, respectively), even though he was not aware of having diabetes mellitus prior to this admission. In addition, this patient had a medical history of hypertension and was previously a smoker. In the following 2 years, the patient had four other hospital admissions due to recurring episodes of cutaneous blistering at the same anatomical location. During the physical examination, multiple clear fluid-filled vesicles of variable dimensions were observed in the left lower limb, surrounded by subcutaneous cellulitis (Fig. 3d). The fluid within bullae was aspirated and cultured; it showed no bacterial growth, consistent with the literature for the diagnosis of bullosis diabeticorum [3, 4].

### Assessments

Peripheral vascular assessment was performed to establish the vascular status in the bilateral lower extremities. Pedal pulses, both the posterior tibialis and the dorsalis pedis artery, were found to be palpable and regular. The left and right ankle brachial index (ABI) was 0.72 and 0.85, respectively. Lower extremity ultrasound revealed atheromatous plaque formation in the tunica intima of the left femoral and popliteal artery, and atherosclerotic plaque formation in the anterior wall of the left femoral artery. Doppler ultrasound demonstrated no vascular abnormalities in major vessels of the left lower limb. Evaluation of the bilateral peripheral arterial system was also achieved with magnetic resonance angiography (MRA). There were no abnormalities in the bilateral internal iliac, external iliac, and femoral artery, except for vessel wall thickening of the left femoral and perforating arteries and enlargement of the left posterior tibial artery. Venography of the left lower limb showed delay in the venous return compared to the contralateral limb. Plain X-ray showed no soft tissue swelling and no bony cortical erosion or periosteal reaction and no subcutaneous gas or osteomyelitis was detected. Combined with the clinical assessments and literature review, recurrent bullosis diabeticorum and cellulitis could be associated with lower limb ischemia and venous stasis. Diabetic neuropathy was excluded through nerve conduct velocity, a gold standard for diagnosis of diabetic neuropathy.

### Preparation of BMMSCs

With reference to our previous research technique [6], 30 ml bone barrow was extracted from the patient under aseptic and anesthetic conditions (Fig. 3a). Mononuclear stem cells were isolated using density gradient centrifugation (Fig. 3b). The harvested autologous BMMSCs, with a total cell count of 7.8 × 10^7^, were cultured with alpha-modified minimum essential medium (α-MEM; Invitrogen-Life Technologies Corp., USA) supplemented with 10% autologous serum. The culture medium was replaced every 3 days. Once approximately 70% confluence was achieved, the cultured stem cells were resuspended in trypsin-EDTA. Cells with an approximately adjusted cell density of 4000 cells per cm^2^ were transferred once every 5 days. The above procedures were done in accordance with the criteria for cell transplantation: (1) negative results of the microbiological tests; (2) endotoxin content of ≤ 5 EU/kg patient body weight; and (3) cell viability of ≥ 95% [6].

### Transplantation procedure and follow-up results

At the same time, the bullae were de-roofed to drain the wound fluid and uncover the underlying wound bed. After which, the wound was dressed with appropriate wound dressing and the patient received combination antibiotics therapy. As we described in a previous study [6], the harvested autologous BMMSCs amount to 8.6 × 10^8^ cells suspended in normal saline after 21 days of culture at three passages (Fig. 3c). Subsequently, the prepared BMMSCs, without cryopreservation, were injected into the left calf muscle with 1 cm intervals after the local infection had subsided (Fig. 3e). Nine months after treatment, the patient developed another episode of bullosis diabeticorum with accompanying cellulitis in the same lower limb and they were successfully treated similarly tot he previous measures. At a 10-year follow-up period, the patient reported no recurrence of bullosis diabeticorum in the left lower limb (Fig. 3f). Peripheral vascular assessment was performed again in October 2017 and the left and right ABI was 1.03 and 0.89, respectively.

## Discussion

Without history of trauma, skin manifestations have been suggested to be probable cutaneous markers for early detection of overt diabetes or prediabetes [1, 8]. As has been reported, bullosis diabecticorum could precede the diagnosis of diabetes mellitus [8], consistent with our reported case. Bullosis diabecticorum is also commonly known as diabetic bullous disease, which is a rare, spontaneous, and non-inflammatory diabetic complication with an incidence rate of 0.16% [9]. The pathogenesis is largely ambiguous, with some researchers believing that a long duration of microvascular disease results in skin dystrophy with capillary basement membrane thickening, which eventually leads to blister formation with tissue hypoxia and microcirculation ischemia [10]. The predisposition to develop bullosis diabeticorum, as we learnt from this case, seems to be associated with varying degrees of limb ischemia. Notably, both Doppler ultrasonography and magnetic resonance angiography showed that this patient had suffered multiple vascular stenoses in the lower extremity. Previous studies have revealed that peripheral arterial disease (PAD) of the lower limb was one of significant and independent risk factors for diabetic foot infection [10, 11]. Therefore, lower extremity cellulitis followed by bullosis diabecticorum was considered as a complication of diabetic PAD in this case.

So far as we know, there is no clinical practice guidance for the appropriate treatment of bullosis diabeticorum. Based on the case findings and literature reports, appropriate management of lower limb ischemia is fundamental for preventing its recurrence. Conventionally, the treatment of severe ischemic lower extremity vascular disease mainly involves both medical and/or early revascularization intervention, either endovascular or open surgery. In some cases, pharmacotherapy alone could not fully address the lower limb ischemia while open surgery or endovascular treatment is contradicted in others due to pre-existing cardiovascular and cerebrovascular diseases. In this case, the patient declined any revascularization treatment but was willing to try conservative treatment. As such, the novel cell therapy was assigned to this patient to eradicate its potential recurrence. Since 2005, our team has been dedicated to the therapeutic use of different stem cells for diabetic wound repair and limb salvage [6, 7, 12–14]. Both BMMSCs and bone marrow-derived mononuclear cells have been used for diabetics with critical limb ischemia and foot ulcer, the former producing better clinical outcomes [6]. In a basic study, we explored the regulatory mechanism of peroxisome proliferator-activated receptor-γ coactivator-1α (PGC-1α) and found that it could enhance engraftment and angiogenesis of mesenchymal stem cells [7]. In another study by Wang et al., the authors observed that PGC-1α also regulates the expression of B-cell lymphoma/leukemia-2 (Bcl-2) and stimulates the survival of mesenchymal stem cells via PGC-1α–ERRα interaction without the influence of serum, hypoxia, and high glucose conditions [15]. In addition, adipose-derived stem cells were also employed for the regulation of diabetic microenvironment [13].

In view of the above theoretical and practical experience, autologous BMMSCs were isolated, cultured, and transplanted into the patient’s left ischemic lower extremities. Other than one reported recurrence at 9 months, bullosis diabeticorum and the associated cellulitis went into remission after transplantation therapy in the following 10 years. In a meta-analysis study by Rigato et al., they showed that cell therapy reduced the risk of amputation by 37% and improved limb salvage by 18% and wound healing by 59%. Cell therapy also significantly increased ABI and transcutaneous oxygen pressure and reduced rest pain [16]. We speculated that the effects of transplanted stem cells might be gradual, starting from cellular differentiation, proliferation, and formation of new capillaries, and eventually improve the lower limb blood flow. At the start of the therapy period, formation of capillary networks and establishment of collateral circulation would not be adequate in ischemic tissue, which may induce another recurrence of bullosis diabecticorum. As blood flow and lower limb ischemia improve, the occurrence slowly tapers off. Thus, stem cell therapy provides an additional, viable option for patients who are ineligible for revascularization procedures. In another meta-analysis study by Fadini et al., the authors reported that traditional revascularization is not suitable for more than one-third of patients and these may benefit from stem cell therapies [17].

## Conclusions

This case describes a patient with a repeated blister disease and cellulitis that were resistant to various treatment modalities but eventually improved with the application of autologous BMMSCs. Autologous bone marrow cell therapy is a feasible, safe, and potentially effective therapeutic strategy for PAD patients who are considered unsuitable candidates for traditional revascularization. This case highlights the potential effect of autologous BMMSCs to relieve repeated cellulitis and blister disease. Early identification of diabetes and its complications and appropriate treatment may improve clinical outcomes and prevent lower limb amputation.

## Abbreviations

ABI: ankle brachial index
Bcl-2: B-cell lymphoma/leukemia-2
BMMSCs: bone marrow mesenchymal stem cells
MRA: magnetic resonance angiography
PAD: peripheral arterial disease
PGC-1α: peroxisome proliferator-activated receptor-γ coactivator-1α
α-MEM: alpha-modified minimum essential medium

## References

[1] Bustan RS, Wasim D, Yderstræde KB, Bygum A (2017). Specific skin signs as a cutaneous marker of diabetes mellitus and the prediabetic state-a systematic review. Dan Med J.

[2] Kurdi AT (2013). Bullosis diabeticorum. Lancet.

[3] Basarab T, Munn SE, McGrath J, Russell JR (1995). Bullosis diabeticorum. A case report and literature review. Clin Exp Dermatol.

[4] Wilson TC, Snyder RJ, Southerland CC (2012). Bullosis diabeticorum: is there a correlation between hyperglycemia and this symptomatology?. Wounds.

[5] Shahi N, Bradley S, Vowden K, Vowden P (2014). Diabetic bullae: a case series and a new model of surgical management. J Wound Care.

[6] Lu D, Chen B, Liang Z, Deng W, Jiang Y, Li S, Xu J, Wu Q, Zhang Z, Xie B, Chen S (2011). Comparison of bone marrow mesenchymal stem cells with bone marrow-derived mononuclear cells for treatment of diabetic critical limb ischemia and foot ulcer: a double-blind, randomized, controlled trial. Diabetes Res Clin Pract.

[7] Lu D, Zhang L, Wang H, Zhang Y, Liu J, Xu J, Liang Z, Deng W, Jiang Y, Wu Q, Li S, Ai Z, Zhong Y, Ying Y, Liu H, Gao F, Zhang Z, Chen B (2012). Peroxisome proliferator-activated receptor-γ coactivator-1α (PGC-1α) enhances engraftment and angiogenesis of mesenchymal stem cells in diabetic hindlimb ischemia. Diabetes.

[8] Lopez PR, Leicht S, Sigmon JR, Stigall L (2009). Bullosis diabeticorum associated with a prediabetic state. South Med J.

[9] Larsen K, Jensen T, Karlsmark T, Holstein PE (2008). Incidence of bullosis diabeticorum -a controversial cause of chronic foot ulceration. Int Wound J.

[10] Peters EJ, Lavery LA, Armstrong DG (2005). Diabetic lower extremity infection: influence of physical, psychological, and social factors. J Diabetes Complicat.

[11] Lavery LA, Armstrong DG, Wunderlich RP, Mohler MJ, Wendel CS, Lipsky BA (2006). Risk factors for foot infections in individuals with diabetes. Diabetes Care.

[12] Jiang XY, Lu DB, Chen B (2012). Progress in stem cell therapy for the diabetic foot. Diabetes Res Clin Pract.

[13] Jiang XY, Lu DB, Jiang YZ, Zhou LN, Cheng LQ, Chen B (2013). PGC-1α prevents apoptosis in adipose-derived stem cells by reducing reactive oxygen species production in a diabetic microenvironment. Diabetes Res Clin Pract.

[14] Wu Q, Chen B, Liang Z (2016). Mesenchymal stem cells as a prospective therapy for the diabetic foot. Stem Cells Int.

[15] Wang M, Yang G, Jiang X, Lu D, Mei H, Chen B (2017). Peroxisome proliferator-activated receptor-γ coactivator-1α (PGC-1α) regulates the expression of B-cell lymphoma/leukemia-2 (Bcl-2) and promotes the survival of mesenchymal stem cells (MSCs) via PGC-1α/ERRα interaction in the absence of serum, hypoxia, and high glucose conditions. Med Sci Monit.

[16] Rigato M, Monami M, Fadini GP (2017). Autologous cell therapy for peripheral arterial disease: systematic review and meta-analysis of randomized, nonrandomized, and noncontrolled studies. Circ Res.

[17] Fadini GP, Agostini C, Avogaro A (2010). Autologous stem cell therapy for peripheral arterial disease meta-analysis and systematic review of the literature. Atherosclerosis.

